# Outbreak of Blastomycosis Among Paper Mill Workers — Michigan, November 2022–May 2023

**DOI:** 10.15585/mmwr.mm735152a2

**Published:** 2025-01-02

**Authors:** R. Reid Harvey, Allyson W. O’Connor, Marcia L. Stanton, Ju-Hyeong Park, Dallas Shi, Perri C. Callaway, Xiaoming Liang, Ryan LeBouf, Rachel Bailey, Ethan Fechter-Leggett, Ian Hennessee, Mitsuru Toda, Rebecca Reik, Mary Grace Stobierski, Jevon McFadden, Sara Palmer, Melissa Millerick-May, Robert Yin, Michael Snyder, Jennifer Meece, Jeremy Olstadt, Alana K. Sterkel, Suzanne Dargle, Olivia Bree, David Weissman, Marie A. de Perio, Stella Hines, Jean Cox-Ganser

**Affiliations:** ^1^Respiratory Health Division, National Institute for Occupational Safety and Health, CDC; ^2^Division of Field Studies and Engineering, National Institute for Occupational Safety and Health, CDC; ^3^Epidemic Intelligence Service, CDC; ^4^Laboratory Leadership Service, CDC; ^5^Division of Foodborne, Waterborne, and Environmental Diseases, National Center for Emerging and Zoonotic Infectious Diseases, CDC; ^6^Michigan Department of Health and Human Services; ^7^Division of State and Local Readiness, Office of Readiness and Response, CDC; ^8^Council of State and Territorial Epidemiologists, Atlanta, Georgia; ^9^Public Health, Delta and Menominee Counties, Escanaba, Michigan; ^10^Marshfield Clinic Research Institute, Marshfield, Wisconsin; ^11^University of Wisconsin-Madison, Madison, Wisconsin; ^12^Wisconsin State Laboratory of Hygiene; ^13^Office of the Director, National Institute for Occupational Safety and Health, CDC.

**Summary**

**What is already known about this topic?**

Blastomycosis is a rare fungal disease often initially misdiagnosed, which can contribute to severe pulmonary illness. Community outbreaks have been reported after soil disruption and outdoor recreational activities in blastomycosis-endemic areas.

**What is added by this report?**

The largest documented outbreak of blastomycosis in the United States occurred among workers at a paper mill in Michigan during 2022–2023. Although environmental sampling did not identify the source of *Blastomyces* exposure in the mill, this was the first recognized blastomycosis outbreak in an industrial, largely indoor setting.

**What are the implications for public health practice?**

Collaboration by local, state, and federal public health authorities with managers and workers can facilitate rapid case detection and implementation of prevention measures to protect workers.

## Abstract

Blastomycosis is a fungal disease caused by inhalation of *Blastomyces* spores from the environment that can result in severe pulmonary illness and high hospitalization rates. In early March 2023, Public Health Delta and Menominee Counties (Michigan) reported a cluster of blastomycosis cases among paper mill workers to the Michigan Department of Health and Human Services (MDHHS). MDHHS subsequently notified CDC. On March 17, paper mill management requested a health hazard evaluation (HHE) from CDC’s National Institute for Occupational Safety and Health (NIOSH) to investigate potential workplace exposures to *Blastomyces* and recommend prevention and control measures at the mill. The workplace epidemiologic investigation combined a NIOSH HHE medical survey consisting of a questionnaire on work and health with *Blastomyces* urine antigen testing of specimens obtained from workers to assist in case finding, with additional case information from MDHHS blastomycosis surveillance data. Assessment of 645 mill workers identified 162 cases of blastomycosis with illness onset during November 1, 2022–May 15, 2023, with the weekly case count peaking at 21 cases in early March 2023. HHE environmental sampling in and around the mill did not identify the source of workers’ *Blastomyces* exposure in the mill. This outbreak was the largest documented blastomycosis outbreak in the United States, and the first associated with a paper mill or an industrial setting. A coordinated public health response facilitated swift prevention measures with recommendations focused on reducing workers’ exposure to *Blastomyces*, including hazard communication, respiratory protection, mill cleaning, and ventilation system improvements.

## Investigation and Results

### Public Health Notification and Response

On February 28, 2023, Public Health Delta and Menominee Counties (PHDM) in Michigan was notified of a cluster of atypical pneumonia cases among workers at a local paper mill in Delta County, Michigan; all patients experienced onset of respiratory symptoms during January–February 2023, and urine antigen testing of patient specimens was positive for *Blastomyces*. PHDM reported the blastomycosis cases to the Michigan Department of Health and Human Services (MDHHS). On March 6, 2023, PHDM notified area health care providers to be alert for additional cases. Within the week, MDHHS notified CDC’s Mycotic Diseases Branch (MDB) and National Institute for Occupational Safety and Health (NIOSH) of eight blastomycosis cases with an additional 14 under investigation; all cases occurred among persons who worked at or visited the paper mill (workers) before illness onset. PHDM, MDHHS, MDB, and NIOSH, in conjunction with mill management and workers, initiated outbreak response and prevention measures.

On March 17, 2023, paper mill management requested a NIOSH health hazard evaluation (HHE) to investigate potential *Blastomyces* exposures and recommend prevention and control measures. NIOSH coordinated four HHE field activities: 1) an initial site visit during March 27–28; 2) an environmental survey and ventilation assessment at the mill during April 24–28; 3) a medical survey, which included a work and health questionnaire and *Blastomyces* urine antigen testing to identify potential cases during April 22–28; and 4) a follow-up environmental survey during August 1–2. This activity was reviewed by CDC, deemed not research, and was conducted consistent with applicable federal law and CDC policy.^†^

### Epidemiologic Investigation

**FIGURE 1:**
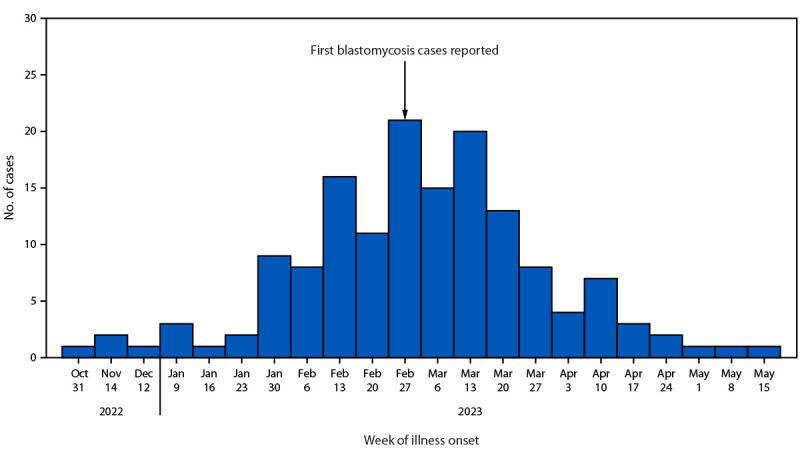
Blastomycosis cases, among workers at a paper mill by week of illness onset (N = 162) — Michigan, November 1, 2022–May 15, 2023* **Abbreviation:** MDHHS = Michigan Department of Health and Human Services. * First blastomycosis cases were reported through Michigan Department of Health and Human Services (MDHHS) surveillance on February 28, 2023; previous cases were identified retrospectively through the National Institute for Occupational Safety and Health medical survey and MDHHS case investigations.

All paper mill employees, contractors, and visitors were invited to participate in the NIOSH medical survey. Among approximately 1,000 workers, 603 participated in the medical survey. Data from an additional 42 mill workers who received a diagnosis of blastomycosis but did not participate in the NIOSH HHE were included in the analysis, coordinated through a data use agreement between NIOSH and MDHHS and voluntary worker consent to share HHE information with public health authorities, resulting in a total of 645 participants. An outbreak case was defined as confirmatory or presumptive^§^ laboratory evidence of blastomycosis or self-reported^¶^ health care provider–diagnosed blastomycosis in a person who worked at or visited the mill during October 1, 2022–July 1, 2023.** Among the 645 workers included in the epidemiologic investigation, 162 (25%) persons with blastomycosis were identified with illness onset during November 1, 2022–May 15, 2023; during the week of February 27–March 5, 2023, the number of new onset cases peaked at 21 (Figure 1). In May 2023, the last case was identified, and on July 1, 2023, the outbreak was declared over. As of April 2024, no additional cases were identified among mill workers. During October 1, 2022–July 1, 2023, one blastomycosis case without an association to the mill was identified in Delta County. Based on 120 blastomycosis cases identified among 603 NIOSH medical survey participants, estimated blastomycosis case prevalence among mill workers was 20%.

### Worker Characteristics

**FIGURE 2:**
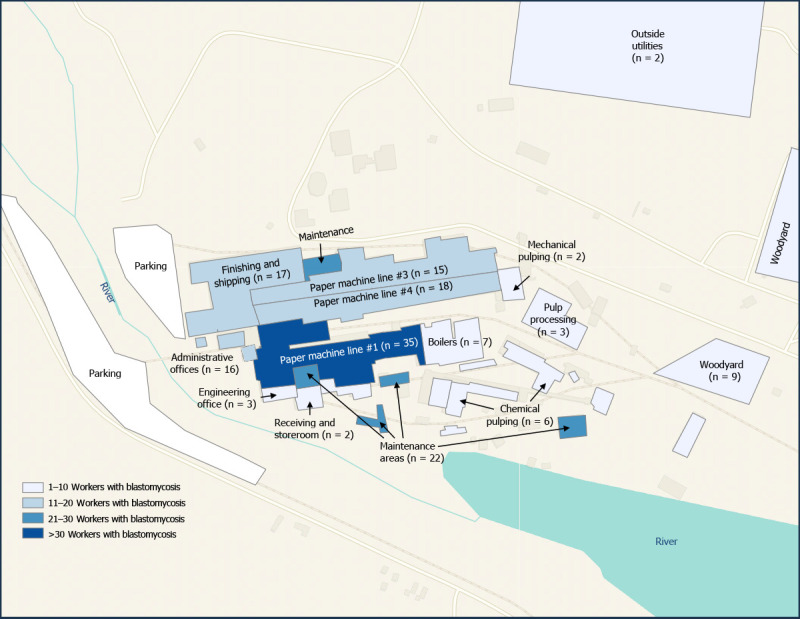
Primary work location* at the paper mill for workers with blastomycosis (n = 162)^†^ — Michigan, November 1, 2022–May 15, 2023 * Map created using ArcGIS Pro. https://www.esri.com/en-us/arcgis/products/arcgis-pro/overview ^†^ Exact number of cases was omitted for some locations to prevent worker identification because number of responses was fewer than five. Primary work location was missing for four workers with blastomycosis.

A majority of workers were men (83%), White (94%), and non-Hispanic (98%), with a median age of 46 years (range = 19–73 years) (Table). Workers with blastomycosis were younger with a shorter tenure at the mill compared with workers without blastomycosis. Among the 645 workers included in the epidemiologic investigation, 162 workers received a diagnosis of blastomycosis and frequently reported signs and symptoms, including cough (90%), shortness of breath (76%), fatigue (76%), and fever or chills (73%); 63% of patients had abnormal lung findings on chest imaging. Among 483 workers without blastomycosis, 294 (61%) reported cough. Eighteen (12%) workers with blastomycosis were hospitalized, and one patient died. Among 573 workers from the NIOSH medical survey with *Blastomyces* urine antigen results, 52 (9%) received a positive test result, 26 (50%) of whom did not report receiving a diagnosis of blastomycosis before the medical survey, including three workers (6%) who reported no signs or symptoms. Based on workers’ primary work locations, paper mill areas with the highest blastomycosis case counts were paper machine line #1 (35) and maintenance (22), although workers in all areas of the mill were affected, including the administrative offices (17) (Figure 2).

**TABLE:** Characteristics of paper mill workers included in a workplace epidemiologic investigation, by blastomycosis case status — Michigan, November 2022–May 2023

Characteristic	No. (%)		
	All workers in workplace epidemiologic investigation N = 645^†^	Blastomycosis case*	
		Yes n = 162^†^	No n = 483^†^
**Demographic**			
Age, yrs, median (range)	46 (19–73)	43 (19–67)^§^	47 (19–73)^§^
**Sex**			
Female	108 (17)	20 (13)	88 (18)
Male	537 (83)	142 (87)	395 (82)
**Race^¶^**			
White	596 (94)	—	—
Other or multiracial	38 (6)	—	—
**Ethnicity^¶^**			
Hispanic or Latino	10 (2)	—	—
Non-Hispanic or Latino	620 (98)	—	—
**Smoking status**			
Never	420 (66)	94 (60)	326 (68)
Former	173 (27)	53 (34)	120 (25)
Current	45 (7)	10 (6)	35 (7)
**Work characteristic**			
Tenure at mill, yrs, median (range)**	8 (0–52)	7 (0–44)^§^	9 (0–52)^§^
**Employment type**			
Employee	603 (94)	147 (92)	456 (95)
Contractor or visitor	39 (6)	13 (8)	26 (5)
**Work type^††^**			
Shift work	397 (63)	100 (65)	297 (62)
Nonshift work	237 (37)	54 (35)	183 (38)
**Department^¶^**			
Administrative offices	95 (15)	20 (13)	75 (16)
Fiberline	46 (7)	14 (9)	32 (7)
Recovery and utilization	39 (6)	—	—
Maintenance and engineering	154 (24)	41 (26)	113 (23)
Paper machine	203 (32)	54 (35)	149 (31)
Finishing and shipping	54 (9)	15 (10)	39 (8)
Wood and coal yard	34 (5)	6 (4)	28 (6)
Other	15 (2)	—	—
**Signs and symptoms and medical findings since October 1, 2022^§§^**			
Cough	437 (68)	143 (90)^§^	294 (61)^§^
Fever, chills, or night sweats	240 (37)	118 (73)^§^	122 (25)^§^
Shortness of breath	224 (35)	123 (76)^§^	101 (21)^§^
Poor appetite or unexpected weight loss	71 (11)	53 (35)^§^	18 (4)^§^
Muscle aches or pain	183 (29)	100 (64)^§^	83 (17)^§^
Fatigue or extreme tiredness	232 (36)	120 (76)^§^	112 (23)^§^
Joint or bone pain**	110 (18)	45 (38)^§^	65 (14)^§^
Skin lesions with no known cause**	28 (5)	10 (8)^§^	18 (4)^§^
Abnormal chest imaging	118 (18)	100 (63)^§^	18 (4)^§^
Hospitalized for blastomycosis	18 (3)	18 (12)	NA
Self-reported respiratory illnesses since October 1, 2022**			
Cold	334 (56)	57 (48)	277 (58)
Influenza	42 (7)	6 (5)	36 (8)
COVID-19	61 (10)	14 (12)	47 (10)
Pneumonia	35 (6)	26 (22)^§^	9 (2)^§^

**Abbreviation:** NA = not applicable.

* An outbreak case was defined as confirmatory or presumptive laboratory evidence of blastomycosis or self-reported health care provider–diagnosed blastomycosis in a person who worked at or visited the paper mill during October 1, 2022–July 1, 2023. Presumptive laboratory evidence was modified from the 2020 Council of State and Territorial Epidemiologists’ surveillance definition for blastomycosis to include positive antigen tests that were below the lower level of quantification.

^†^ This value is the maximum number of participants; number of participants varies because of missing data.

^§^ A value of p<0.05 indicates statistically significant values. Wilcoxon signed-rank tests were used for continuous variables, and Pearson chi-square tests were used for categorical variables to test differences by the case classification.

^¶^ Number and percentage omitted to avoid worker identification because of small numbers of responses (fewer than five) in at least one cell. Persons of Hispanic or Latino (Hispanic) origin might be of any race but are categorized as Hispanic; racial groups are non-Hispanic.

** Only National Institute for Occupational Safety and Health medical survey participants answered these questions (603); percentages are calculated among 120 workers for those who met the case definition for blastomycosis. Information from the 42 patients on the Michigan Department of Health and Human Services line list were excluded.

^††^ Shift work included rotating day and night work shifts. Nonshift work had regularly set workdays (e.g., Monday–Friday).

^§§^ Medical findings also included inflammation of the brain, such as meningitis or encephalitis, or a focal brain lesion; abscess, granuloma, or lesions in other parts of the body besides the skin; and bone or joint abnormality, such as a bone infection or a pathologic fracture. Results were not reported for these findings because of small numbers of responses.

### Environmental Assessment

The paper mill is located adjacent to a river and occupies 2,200 acres, including the indoor mill buildings, outdoor woodyards, water treatment lagoons, and landfill. Mill buildings contained approximately 400 heating, ventilation, and air conditioning (HVAC) systems. The ventilation assessment involved visual assessment of the air handling units and makeup air units (these units supply fresh air from outside the building into the occupied space). Among the 67 units assessed, 59 (88%) were makeup air units providing unfiltered outdoor air into the mill; many had dirt and organic debris observed in the coils.

The Marshfield Clinic Research Institute (https://marshfieldresearch.org/) and the University of Wisconsin-Madison analyzed environmental samples for *Blastomyce*s by both polymerase chain reaction (PCR) and culture for identification. Environmental samples from the mill collected during the HHE included soil, wood chips, indoor surface dust, and water, dust, duct lining, and filters from the HVAC systems. Additional environmental samples were collected during August 1–2, 2023, while excavation activities took place for a bridge being constructed over the river near the mill. Among 533 indoor and outdoor environmental samples analyzed, no *Blastomyces* was detected by PCR or culture.

## Public Health Response

Beginning April 17, 2023, paper mill management voluntarily idled production for 3 weeks, for ventilation ductwork cleaning and upgrading filters in all air handling units. During April 20–21, NIOSH, MDB, PHDM, and MDHHS staff members conducted 10 sessions for workers to provide information about blastomycosis and the NIOSH HHE. Mill managers facilitated training for workers regarding potential hazards and associated safe practices, procedures, and protective measures to reduce *Blastomyces* exposure and encouraged workers to report potential blastomycosis symptoms to health care providers. During the outbreak, NIOSH recommended that workers wear fit-tested NIOSH-approved N95 respirators,^††^ especially those workers at risk for severe disease (e.g., persons who were immunosuppressed) or performing potentially high-risk activities (e.g., changing HVAC filters or disturbing soil). NIOSH communicated HHE findings and recommendations to paper mill managers, workers, and public health partners through routine conference calls and interim letters.

## Discussion

Blastomycosis is a rare infectious disease caused by inhalation of *Blastomyces* fungal spores from the environment. Pulmonary illness is most common, ranging from mild, self-limited respiratory symptoms to severe, potentially fatal pneumonia; approximately 25% of cases include extrapulmonary disease, often involving skin lesions (*1*). As many as one half of people infected with *Blastomyces* remain asymptomatic (*2*). Diagnostic and treatment delays are common because of clinical similarities with other respiratory infections, although antifungal medication is important to prevent progression or recurrence of disease in symptomatic patients (*1*). *Blastomyces* is found in moist soil or decaying wood and leaves in the midwestern and southeastern United States, often near rivers and lakes. This outbreak was the largest documented blastomycosis outbreak ever reported in the United States. Although work-related blastomycosis has been reported in industries such as farming, construction, and landscaping (*3*), this report investigates the first recognized blastomycosis outbreak associated with a paper mill or other industrial worksite. The mill’s location along a riverway in a wooded area is consistent with *Blastomyces’* habitat^§§^; however, the specific environmental factors in or around the mill that led to this outbreak remain unknown.

*Blastomyces* typically does not propagate indoors (*4*); however, spores potentially infiltrated mill buildings through unfiltered ventilation systems or open bay doors, leading to indoor fungal exposure, likely over several months. During a 2019 outbreak in Wisconsin, *Blastomyces* was detected by PCR from indoor air samples (*5*). Because of the challenges in identifying *Blastomyces* from the environment (*1*), the lack of positive samples from the mill does not rule out the presence of *Blastomyces*. Given the 2-week to 3-month incubation period for blastomycosis (*6*), *Blastomyces* exposure at the mill likely began as early as mid-October 2022 and extended through at least February 2023, and as late as April 2023.

Blastomycosis is an endemic and reportable disease in Michigan, with 186 cases reported during 2007–2017 (mean annual incidence = 0.2 cases per 100,000 population) (*7*). In Delta County, fewer than one blastomycosis case was reported annually; the single non-mill–related case identified during the workplace outbreak was consistent with previous surveillance. Blastomycosis might be underreported in Delta County; some northern counties in neighboring Wisconsin with similar environments have an annual reported incidence exceeding 20 cases per 100,000 population (*7*). Urine antigen testing in the NIOSH medical survey was useful for identifying workers with potentially undiagnosed blastomycosis. Case finding through HHE using urine antigen testing identified three asymptomatic patients. In addition, approximately one half of workers with positive urine test results did not report blastomycosis diagnoses, indicating that urine antigen testing helped to identify cases not included in MDHHS surveillance data.

Twelve percent of workers with blastomycosis were hospitalized in this outbreak; previous reports indicate that approximately 65% of reported patients with blastomycosis require hospitalization (*8*). Early outbreak detection enabled active case finding and directed public health messages, urging symptomatic workers to seek medical care promptly. Despite a relatively healthy workforce, respiratory symptoms, particularly cough, were common in workers without blastomycosis and were potentially attributed to other respiratory illnesses, during the 6-month exposure window that spanned winter. Conditions associated with paper milling, including generation of and exposure to paper dust (*9*), indoor dampness, and fungi other than *Blastomyces* (*10*) might also have contributed to respiratory symptoms among mill workers.

Challenges of the investigation included the difficulties identifying *Blastomyces* from environmental samples (*1*) and the timing of the NIOSH HHE medical survey, particularly the urine antigen screening, given the variable incubation period for blastomycosis (*6*). In addition, most workers who self-reported blastomycosis had been prescribed antifungal medication from their health care provider, which could have affected urine antigen test results from the NIOSH medical survey. Paper mill managers acted quickly after outbreak recognition to engage public health authorities and implement prevention measures. Rapid and coordinated public health actions by local, state, and federal public health authorities and information sharing among partners enabled a swift response to protect mill workers.

### Implications for Public Health Practice

Industries and occupations that routinely perform outdoor work activities such as disturbing soil in areas with endemic blastomycosis might consider providing worker training and education to enhance awareness of *Blastomyces* and reduce the likelihood of work-related blastomycosis among workers. Although not specific to *Blastomyces*, routine HVAC maintenance, including changing filters, fan belts, and other parts, along with cleaning the system when necessary and employing housekeeping best practices might help reduce the likelihood of *Blastomyces* propagating indoors in areas where the disease is endemic. Work-related exposure to *Blastomyces* might be considered by health care providers and public health authorities in these areas to detect future outbreaks early and implement public health interventions quickly.

## Footnotes

* These authors contributed equally to this report.

† 45 C.F.R. part 46, 21 C.F.R. part 56; 42 U.S.C. Sect. 241(d); 5 U.S.C. Sect. 552a; 44 U.S.C. Sect. 3501 et seq.

§ Presumptive laboratory testing from 2020 Council of State and Territorial Epidemiologists (CSTE) case definition for blastomycosis was modified to include positive *Blastomyces* antigen tests that were below the lower level of quantification. https://cdn.ymaws.com/www.cste.org/resource/resmgr/2019ps/final/19-ID-02_Blastomycosis_final.pdf

¶ A self-reported diagnosis of blastomycosis from a health care provider since October 1, 2022, on the NIOSH medical survey met the case definition, because some cases were not reported to MDHHS through surveillance mechanisms. Many workers with self-reported blastomycosis who were not known to MDHHS as probable or confirmed cases from the community epidemiologic investigation were taking antifungal medications at the time of the medical survey.

** MDHHS and PHDM defined an outbreak case as an illness that met the 2020 CSTE clinical and laboratory criteria for confirmed or probable blastomycosis cases in a person who lived in or had spent ≥40 hours in Delta County, Michigan since September 1, 2022, and whose illness onset occurred during December 1, 2022–July 1, 2023. MDHHS and PHDM identified 131 cases through blastomycosis surveillance in this outbreak.

†† N95 and NIOSH-approved are certification marks of the U.S. Department of Health and Human Services registered in the United States and several international jurisdictions.

§§
https://www.cdc.gov/blastomycosis/causes/?CDC_AAref_Val
